# Profiling of 179 miRNA Expression in Blood Plasma of Lung Cancer Patients and Cancer-Free Individuals

**DOI:** 10.1038/s41598-018-24769-2

**Published:** 2018-04-20

**Authors:** Ivan A. Zaporozhchenko, Evgeny S. Morozkin, Anastasia A. Ponomaryova, Elena Y. Rykova, Nadezhda V. Cherdyntseva, Aleksandr A. Zheravin, Oksana A. Pashkovskaya, Evgeny A. Pokushalov, Valentin V. Vlassov, Pavel P. Laktionov

**Affiliations:** 1 Laboratory of Molecular Medicine, SB RAS Institute of Chemical Biology and Fundamental Medicine, Novosibirsk, Russia; 2 Meshalkin National Medical Research Center, Ministry of Health of the Russian Federation, Novosibirsk, Russia; 3 Laboratory of Molecular Oncology and Immunology, RAMS Tomsk Cancer Research Institute, Tomsk, Russia; 4 Department of Applied Physics, National Research Tomsk Polytechnic University, Tomsk, Russia; 5 Department of engineering problems in ecology, Novosibirsk State Technical University, Novosibirsk, Russia; 6 Laboratory for Translational Cell and Molecular Biomedicine, National Research Tomsk State University, Tomsk, Russia

## Abstract

Lung cancer is one of major cancers, and survival of lung cancer patients is dictated by the timely detection and diagnosis. Cell-free circulating miRNAs were proposed as candidate biomarkers for lung cancer. These RNAs are frequently deregulated in lung cancer and can persist in bodily fluids for extended periods of time, shielded from degradation by membrane vesicles and biopolymer complexes. To date, several groups reported the presence of lung tumour-specific subsets of miRNAs in blood. Here we describe the profiling of blood plasma miRNAs in lung cancer patients, healthy individuals and endobronchitis patients using miRCURY LNA miRNA qPCR Serum/Plasma Panel (Exiqon). From 241 ratios differently expressed between cancer patients and healthy individuals 19 miRNAs were selected for verification using the same platform. LASSO-penalized logistic regression model, including 10 miRNA ratios comprised of 14 individual miRNAs discriminated lung cancer patients from both control groups with AUC of 0.979.

## Introduction

Lung cancer (LC) is causing more than 1.3 million deaths worldwide annually^1^. Early detection of lung cancer is critical for survival, as indicated by the more than tenfold (4 vs 54%) difference in the 5-year survival rate between early stage LC and progressed disease^2^. Screening and diagnosis of lung cancer is generally based on chest radiography, computed tomography, and magnetic resonance imaging, aided by histopathological examination of resected tumour tissue and material from bronchoscopy biopsies, fine needle aspirations or sputum in order to confirm the diagnosis and determine the tumour subtype. Unfortunately, current methods fail to reliably detect localized early stages and most patients are diagnosed with advanced disease that cannot be effectively treated locally^3,4^. Despite recent advancements in screening applications of low dose computed tomography (LDCT), an emerging contender for LC screening technique, the number of incidentally found benign lung nodules exceeds cancer diagnoses by a factor of 40^5^. The situation is further complicated by the high intratumour heterogeneity and general histological diversity of lung malignancies^3^.

Study of tumour genetics and molecular mechanisms of cancer have opened the door to the molecular cancer diagnostics^6^. So far, the results are promising, but most developed tests are based on invasive sample acquisition techniques (e.g. Epi proLung by Epigenomics AG that uses bronchial lavage), creating a barrier for their routine use in LC diagnosis and prohibiting their application for LC screening. On the other hand, circulating blood is firmly established as a lucrative source of lung cancer biomarkers, due to the high blood perfusion of the tumours^7^. Previously, tumour-derived nucleic acids were found circulating in the bloodstream of cancer patients^8^. Of particular interest are the cell-free miRNAs – small non-coding RNAs that regulate expression of a significant part of the genome, including genes involved in malignant transformation^9^. Several properties of miRNAs suggest them as possible candidate biomarkers for LC. To date, a plethora of studies showed that miRNAs are frequently (and often drastically) deregulated in most human cancers, including lung cancer, and shifts in miRNA expression are considered one of the characteristic features of malignant transformation^9^. It is also known that miRNAs can travel in bodily fluids for extended periods of time, shielded from degradation by membrane vesicles and biopolymer complexes^10–13^. This layer of protection plays a critical role in defining their stability and accessibility as disease biomarkers. So far, a number of groups have reported specific subsets of circulating miRNAs in blood that were linked to tumour location, clinical properties, and genetic phenotypes, including certain EGFR mutations and ALK fusion positivity^10,14–16^. Even more importantly, miRNAs have been proposed as indicators of disease progression, metastasis, patient prognosis and survival^17–19^. However, in spite of numerous reports showing the potential of circulating miRNAs in the diagnosis of LC, to our knowledge only two blood-based miRNA panels have successfully progressed to clinical testing as complementary tests for LDCT^20,21^. However, in view of these success stories there are still many gaps in our knowledge regarding both the features of miRNA biology in cancer and the key technical aspects of their use as biomarkers.

Previously we have developed a method for isolation of circulating miRNA from blood and validated it using a panel of candidate biomarker miRNAs^22,23^. In the present study we investigated the expression of 179 cell-free miRNAs in blood plasma of non-small cell lung cancer patients and healthy individuals in order to identify potential markers for lung cancer diagnostics.

## Results

### Study design

**Figure 1:**
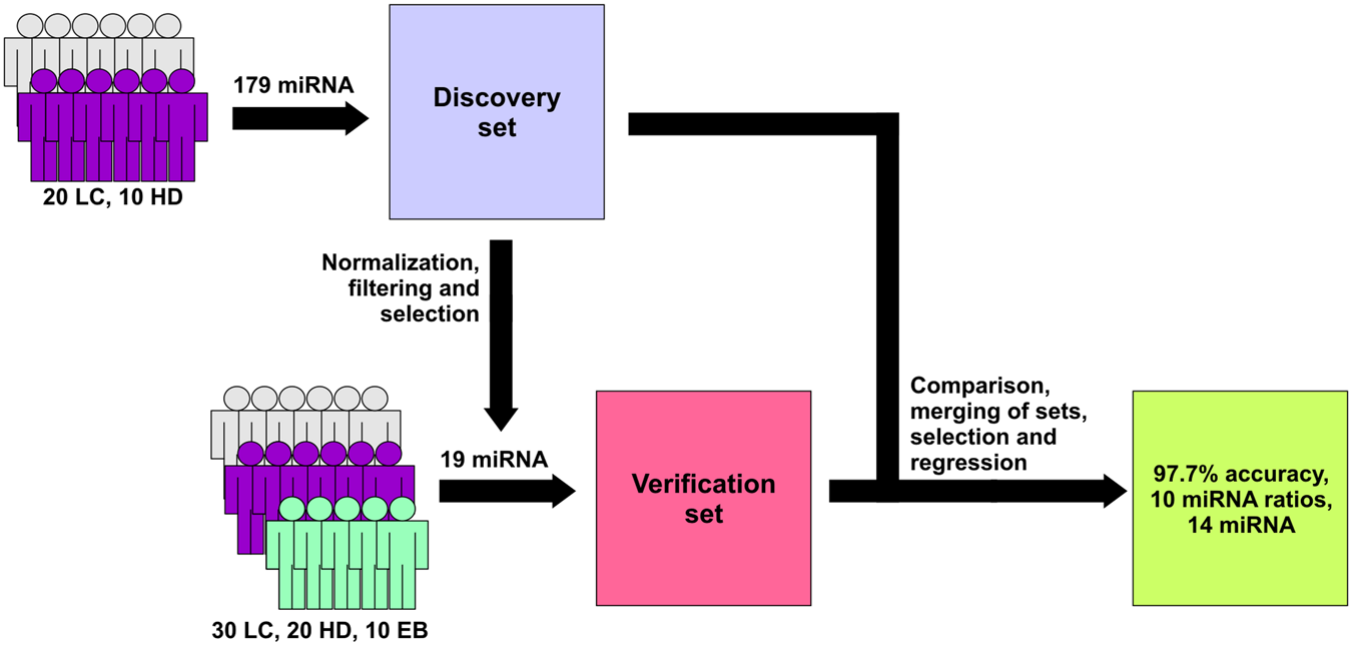
Overview of the Study Design. LC – lung cancer patients, HD- healthy individuals, EB – patients with endobronchitis.

In this study, we have attempted to investigate the pool of miRNAs, circulating in blood plasma of lung cancer patients, in search of biomarkers for detection of lung cancer (Fig. 1). Non-small cell lung cancer patients diagnosed with squamous cell carcinoma (SCC) and adenocarcinoma (AD) were enrolled in the study, and were analysed either individually or as a combined lung cancer (LC) group. In addition to cancer patients, study population also included a non-cancerous lung disease control group comprised of individuals diagnosed with hyper- or metaplastic endobronchitis (EB) and cancer-free group of healthy volunteers (HD). To isolate plasma miRNA we employed an original phenol-free extraction technique used in our groups’ previous reports. For profiling of miRNA the miRCURY LNA qPCR platform was chosen as it previously compared favourably to other options of measuring miRNA expression in samples with low RNA content, such as blood plasma and serum^24^. At the first phase of the study, the data of miRNA expression in SCC, AD and HD groups was obtained and a subset of 19 miRNAs was selected for further study. Next, additional information about the expression of these miRNAs was obtained using an independent sample of LC patients, HD and EB samples. Finally, LASSO penalized regression model based on miRNA ratios was fitted using the data from both data sets.

### Profiling in discovery set

Small RNAs were isolated from blood plasma of 20 LC patients (14 SCC and 6 AD) and 10 healthy individuals (HD). Profiles of miRNA expression were obtained using miRCURY LNA miRNA qPCR Serum/Plasma panel (Exiqon) incorporating assays for 179 miRNAs most commonly found in peripheral blood. The presence of miRNA in all samples was assessed by qRT-PCR for miR-16 and -126 before shipping and upon arrival at Exiqon by the internal quality control. All samples passed internal QC checks for reverse transcription and qPCR efficiency, although several samples had elevated haemolysis scores, and one sample (H02) had a particularly high score (Supplementary Fig. S1). The data obtained after profiling of these samples were the discovery dataset.

**Figure 2:**
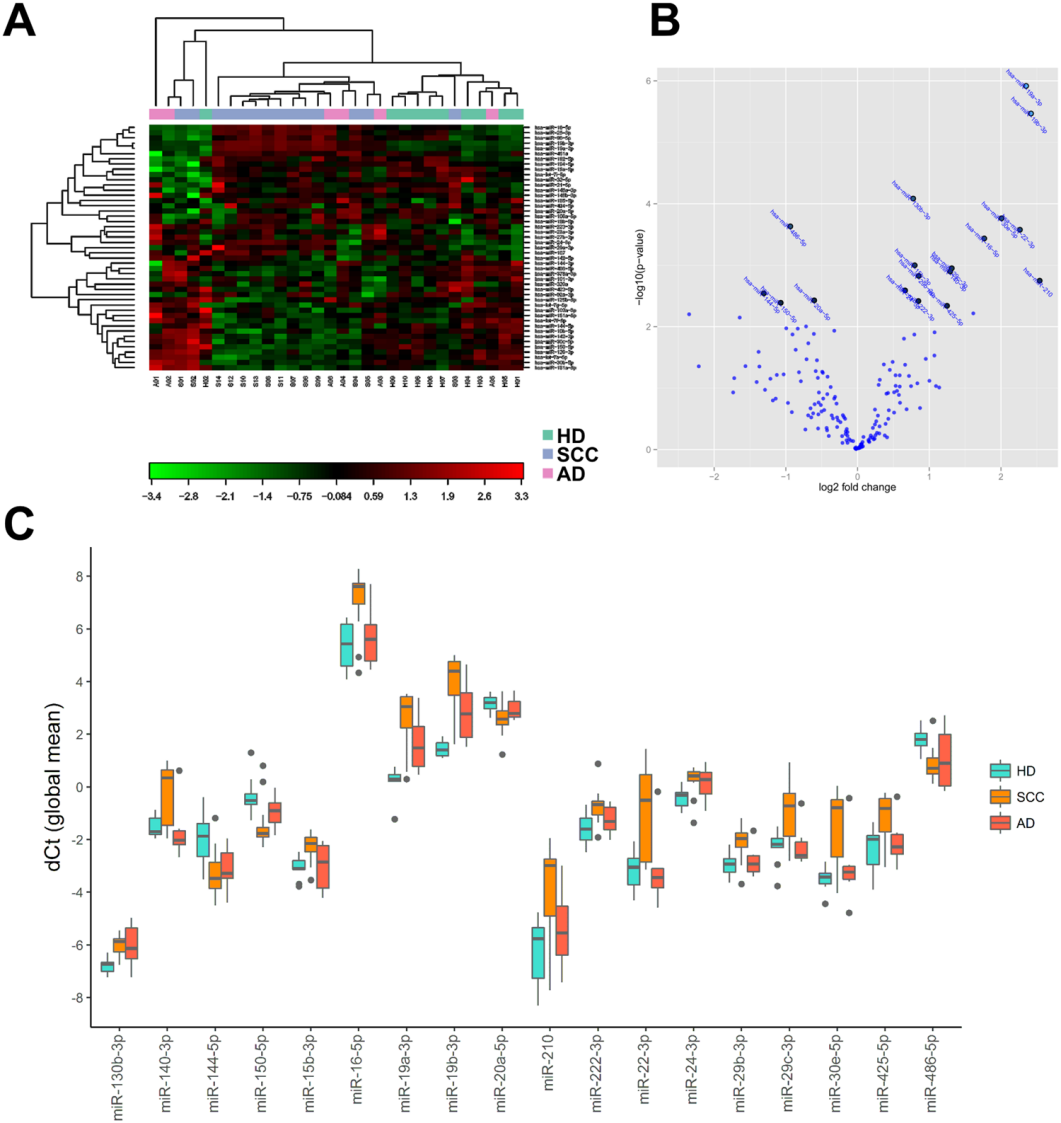
Profiling of plasma miRNA expression in discovery set. (**A**) Heatmap and unsupervised clustering of miRNAs with most variable expression in all samples; (**B**) Volcano plot of top miRNAs differentiating SCC and HD; (**C**) Box plots of differently expressed miRNAs. Global mean normalized miRNA expression.

Exploratory analysis of the discovery set identified significant differences in the expression of subsets of miRNAs (Fig. 2). Although principal component analysis did not identify any meaningful clustering of samples by group, unsupervised clustering showed that most samples were gravitating towards one of two groups - cancer and non-cancer, without any regard for cancer subtype. An exception to this rule was a group of five samples on the left side of the plot, consisting of samples from all three groups, which shared some similarity to the non-cancer group. Pairwise comparisons between all three groups showed that 18 miRNAs were significantly different between SCC patients and healthy individuals according to pairwise comparisons of miRNA expression (T-test, p < 0.05, Benjamini-Hochberg correction) (Table 1). At the same time, no differences in miRNA expression between the AD and either SCC or HD groups passed the significance threshold. Based on the lack of observed differences in either analysis, SCC and AD patients were merged into a combined LC group for all further analyses.

**Table 1:** List of miRNAs significantly differently expressed between patients with squamous cell carcinoma (SCC) and healthy individuals (HD).

miRNA	Mean dCq		SD		Fold change	P-value	Adjusted P-value
	SCC	HD	SCC	HD			
hsa-miR-19a-3p	2.6	0.24	1.1	0.56	5.1	0.0000012	0.00020
hsa-miR-19b-3p	3.9	1.4	1.2	0.30	5.3	0.0000034	0.00029
hsa-miR-130b-3p	−6.0	−6.8	0.43	0.30	1.7	0.000082	0.0046
hsa-miR-30e-5p	−1.5	−3.5	1.5	0.46	4.0	0.00017	0.0072
hsa-miR-486-5p	0.85	1.8	0.62	0.43	−1.9	0.00023	0.0074
hsa-miR-22-3p	−0.93	−3.2	1.7	0.79	4.8	0.00026	0.0074
hsa-miR-16-5p	7.1	5.4	1.2	0.89	3.4	0.00037	0.0088
hsa-miR-15b-3p	−2.3	−3.1	0.52	0.45	1.7	0.0010	0.021
hsa-miR-29c-3p	−0.96	−2.3	1.1	0.66	2.5	0.0011	0.021
hsa-miR-140-3p	−0.19	−1.5	1.2	0.41	2.4	0.0013	0.021
hsa-miR-29b-3p	−2.1	−2.9	0.69	0.47	1.8	0.0015	0.023
hsa-miR-210	−3.8	−6.3	1.6	1.3	5.8	0.0018	0.025
hsa-miR-24-3p	0.26	−0.41	0.57	0.39	1.6	0.0026	0.033
hsa-miR-144-5p	−3.2	−1.9	0.89	0.94	−2.5	0.0029	0.034
hsa-miR-20a-5p	2.6	3.2	0.59	0.30	−1.5	0.0037	0.040
hsa-miR-222-3p	−0.72	−1.6	0.64	0.57	1.8	0.0038	0.040
hsa-miR-150-5p	−1.5	−0.38	0.88	0.74	−2.1	0.0041	0.040
hsa-miR-425-5p	−1.2	−2.4	0.95	0.87	2.4	0.0046	0.043
hsa-miR-324-5p	−5.5	−7.1	0.84	0.94	3.1	0.0061	0.053
hsa-miR-133a	−8.2	−5.8	1.3	2.0	−5.1	0.0063	0.053

### Comparison of the results to The Cancer Genome Atlas project data

In order to test, whether the results comply with any known patterns of miRNA expression in lung cancer tissues we have looked into the publicly available data. The miRNA-Seq data from tumour tissues and adjacent normal lung tissues of 45 SCC patients enrolled in the TCGA-LUSC project was retrieved and the expression of deregulated miRNAs, identified at the previous step, was compared between the two sets.

**Figure 3:**
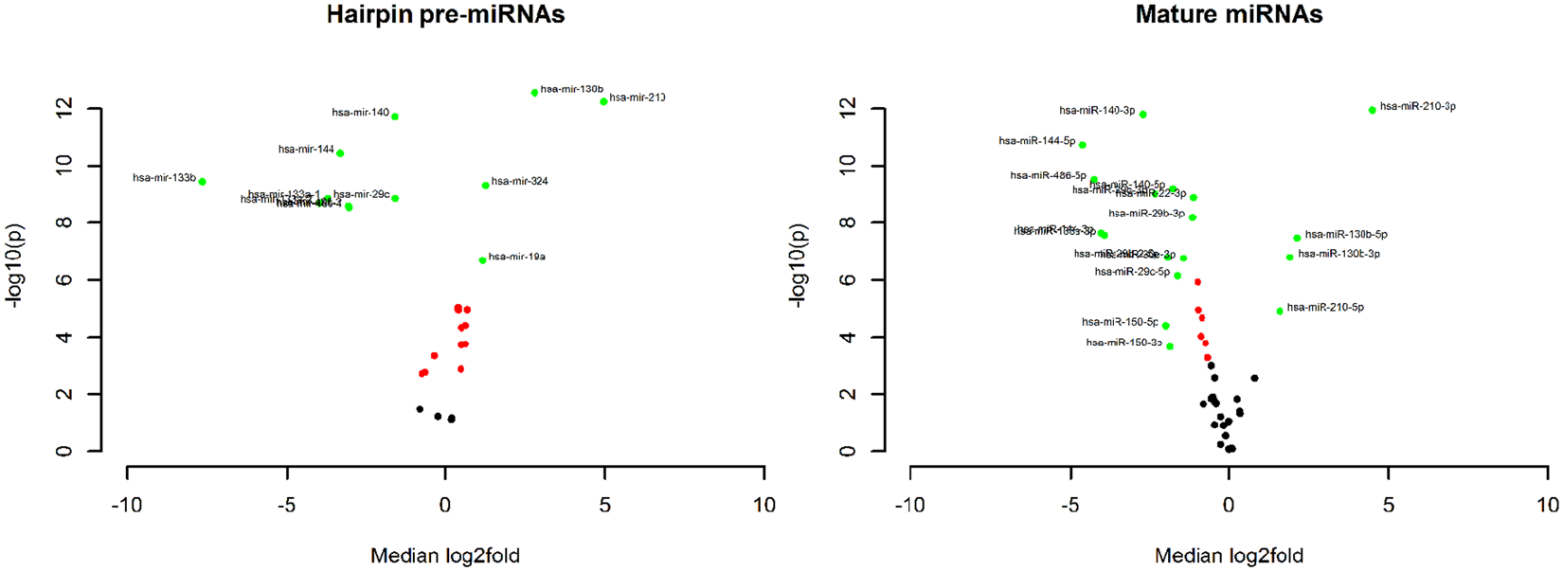
Volcano plots of pre-miRNA and mature miRNA expression in matched tumour and adjacent normal tissue of squamous cell carcinoma patients. TCGA-LUSC project miRNA-Seq data, RPM normalized. Median log2 difference in expression between matched tumour and normal tissue samples.

Using paired comparison statistics, we found that some of miRNAs in Table 1 that were deregulated in plasma of SCC patients were also deregulated in the lung tumours of patients enrolled in the TCGA-LUSC project, which suggests that tumours may be the primary source of variation for the levels of these miRNAs in the bloodstream (Fig. 3). For miR-210 and -130b significant upregulation of expression in tumour tissue was discovered at both pre-miRNA and mature miRNA levels, while for miR-19a, -19b, 15b and miR-324 only the changes in hairpin expression were found. Similarly, miR-144, -150, -29b, -29c, -486, -22 were downregulated at both levels, while only mature miR-30e and -425 were downregulated.

### Ratio-based statistical analyses of miRNA expression

**Figure 4:**
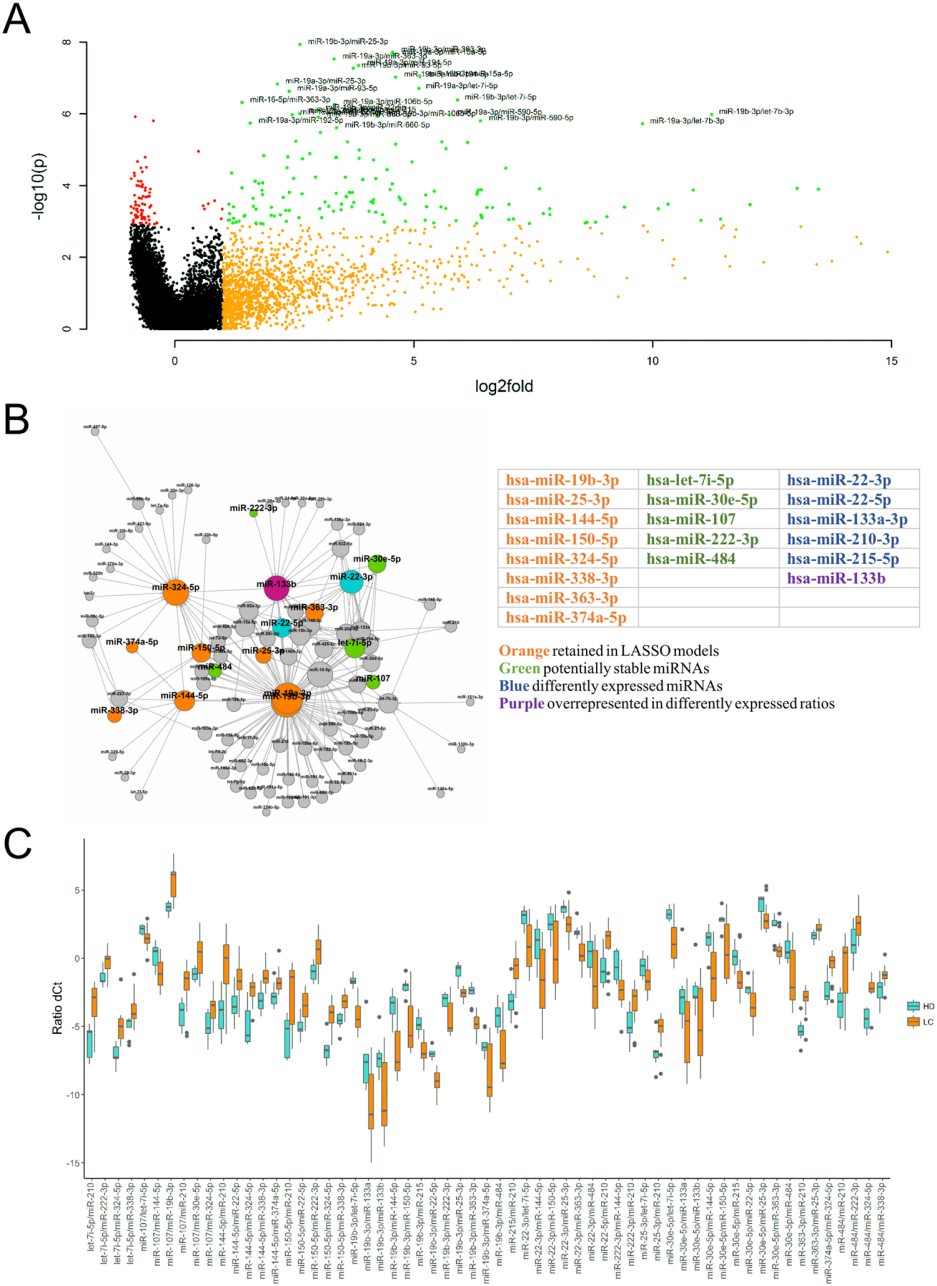
Analysis of miRNA expression after ratio-based normalization. (**A**) Volcano plot of significant miRNA ratios. *Red* – significant at 0.05 after Benjamini-Hochberg correction, *orange* – median fold change between cancer and healthy groups >1 (dCt difference >2), *green* – both. Names are provided for ratios at p < 0.001; (**B**) Network graph of miRNAs in differently expressed ratios (Kamada-Kawai); (**C**) Boxplots of ratios with significantly different expression in discovery set comprised of 19 selected miRNAs.

Following the exploratory analyses, we have looked at how discovery set data can be verified. Reproducing panel expression data can be problematic due to the lack of stable normalizers to properly relate the expression for circulating miRNAs and act as a substitute for the stable global mean normalization. To circumvent this issue ratio based normalization was applied to all miRNAs with call rates higher than 80% resulting in a total of 9729 miRNA ratios. One sample with low miRNA counts and/or potential haemolysis was eliminated from further analysis (H02). Statistical comparison using two-way ANOVA identified 241 ratios (98 individual miRNAs) with significantly different expression between HD and the combined LC group (p < 0.05, Benjamini-Hochberg correction) (Fig. 4A,B).

Unsurprisingly, many of the miRNAs found in Table 3, such as miR-19a, -19b, -144, 150, 22-3p and -16 were highly represented in the ratios with significantly different expression, as indicated by Fig. 4B. However, few miRNA, not previously in the analysis were also common, including miR-133b, let-7b and let-7i, which may indicate their role as relatively stable miRNAs not concerned with lung tumorigenesis. Notably, miR-324 was also often found in differently expressed ratios, despite the low significance level of its expression in respect to global mean.

One disadvantage of using ratio-based normalization on large sets of miRNAs is that the number of significant predictors can be artificially inflated by the presence of multiple ratios with high collinearity. To reduce the complexity of the data we used a combination of different tools. Firstly, we used LASSO-penalized regression to identify minimal subsets of miRNA ratios that discriminate between cancer and non-cancer groups with absolute specificity and sensitivity. Secondly, we looked at several recent reports of haemolysis-related miRNAs^25,26^ and performed manual filtering of miRNAs tentatively associated with haemolysis in stringent and semi-stringent modes (Supplementary Table S1). After reviewing the composition of models resulting from filtered and non-filtered ratio sets, also taking into account the magnitude of observed differences (no less than 1 Cq mean difference between two ratios), seven miRNA ratios were identified as best predictors of cancer (Supplementary Fig. S2).

Based on the accumulated data we have selected 19-miRNAs for verification, including miRNAs from ratios comprising best performing regression models while also keeping the number of significant miRNA connections maximized. Additionally, several individual miRNA markers identified in the exploratory phase and a set of stably expressed miRNAs were included in the panel to serve as a measure of quality control (Fig. 4B).

### Verification of miRNA expression

Expression of the 19 selected miRNAs was measured in an independent sample of LC patients, healthy individuals (HD) and patients diagnosed with hyper- and metaplastic endobronchitis (EB) using custom miRCURY LNA miRNA qPCR Pick & Mix panel. Samples were tested for haemolysis and presence of miRNA prior to shipping by performing qRT-PCR for miR-23a and miR-451a. Only samples with miR-23a/miR-451a ratio of dCq < 7.5 were analysed. These data comprised the verification dataset (Supplementary Dataset 1).

**Figure 5:**
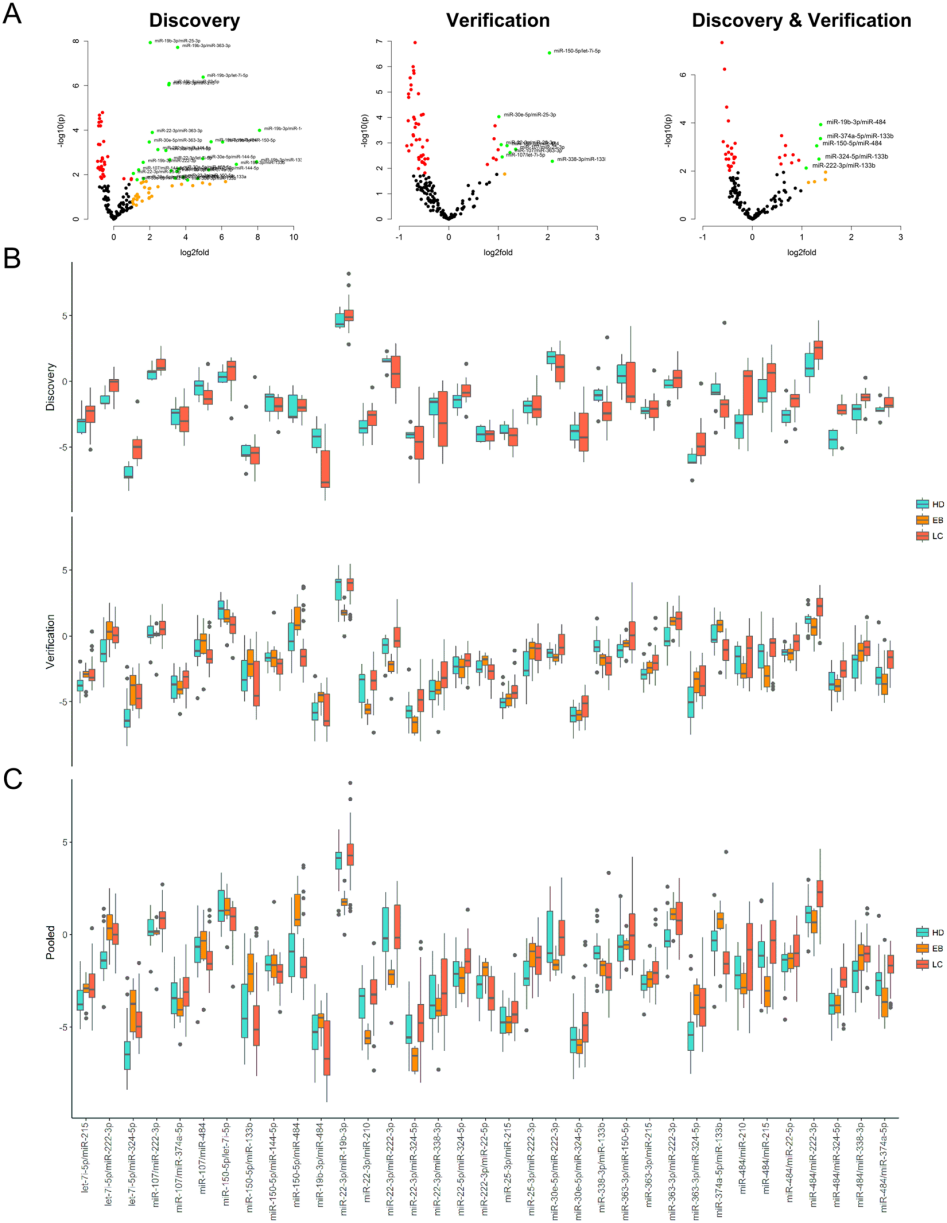
Boxplots of miRNAs deregulated in both sets. (**A**) Volcano plots of miRNA expression for ratios significantly deregulated in both discovery and verification sets and pooled data; (**B**) Expression of miRNA ratios in discovery set and verification sets; (**C**) Expression of miRNA ratios in pooled data.

Ratio-based normalization was applied to the data and expression of miRNA ratios was compared between the groups. At large, the differences between the groups were less pronounced than in the original experiment. Nevertheless, pairwise comparisons showed that 26 ratios were different between LC and HD, 29 were different between EB and HD and 56 were different between LC and EB patients (ANOVA, p < 0.05, Benjamini-Hochberg correction), thus indicating that all three groups were characterized by distinct profiles of plasma miRNA expression (Fig. 5A,B, Supplementary Dataset 2). Set of 20 ratios in Table 2 had significantly different expression between LC and both EB and HD control groups (ANOVA, p < 0.05, Benjamini-Hochberg correction).

**Table 2:** Ratios with significant difference between LC and non-cancer groups in verification set.

Ratio	P	P adjusted	Fold change
miR-150-5p/let-7i-5p	2.27E-05	0.003888	−1.02401
miR-150-5p/miR-144-5p	0.000218	0.009329	−0.87207
miR-484/miR-222-3p	0.000111	0.009329	1.094977
miR-484/miR-324-5p	0.00019	0.009329	1.079881
miR-22-3p/miR-222-3p	0.000531	0.009413	1.254322
miR-30e-5p/miR-222-3p	0.000373	0.009413	0.728391
miR-363-3p/miR-150-5p	0.000309	0.009413	1.142667
miR-25-3p/miR-150-5p	0.000588	0.009413	1.176
miR-25-3p/miR-215	0.000606	0.009413	0.801954
miR-484/miR-374a-5p	0.000518	0.009413	1.189276
miR-374a-5p/miR-133b	0.00057	0.009413	−1.3027
miR-22-3p/miR-324-5p	0.000844	0.012032	1.233095
miR-484/miR-338-3p	0.001091	0.014354	1.051429
miR-363-3p/miR-215	0.001563	0.019091	0.788609
miR-150-5p/miR-484	0.002034	0.023183	−1.42567
miR-30e-5p/miR-324-5p	0.002371	0.025336	0.768519
miR-22-3p/miR-338-3p	0.00282	0.028369	1.146786
miR-22-3p/miR-374a-5p	0.004404	0.041843	1.305524
miR-484/miR-22-5p	0.004784	0.043059	0.582635
miR-363-3p/miR-222-3p	0.005289	0.045222	0.821805

We also looked at the consistency of changes in miRNA expression across the two experiments by comparing the sets of significantly deregulated miRNA ratios. Several miRNAs, notably the miR-19a and -19b, and several low abundance miRNAs, exhibited inconsistent expression, which affected the performance of ratios including these miRNAs. Overall, 35 ratios consistently deregulated in both sets were selected for further analysis (Fig. 5). Under the assumption that there were no significant differences in the experimental conditions the data from both experiments were pooled together. This step was additionally justified by the Kolmogorov-Smirnov tests indicating no difference in distribution of miRNA expression between the two datasets in either HD or LC groups. Figure 5C displays the expression of selected miRNA ratios in the combined dataset.

**Figure 6:**
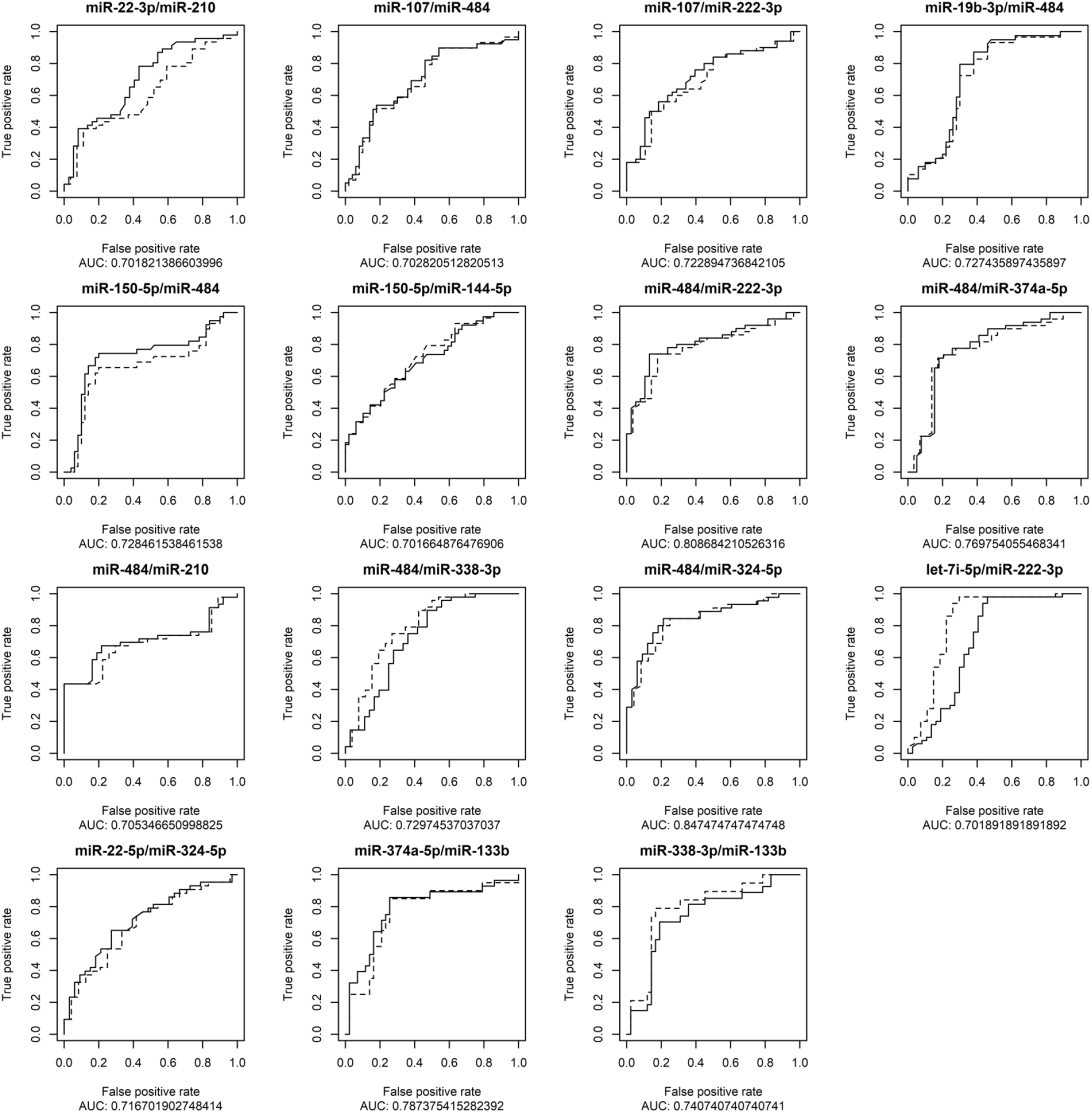
ROC curves of most deregulated miRNAs. Solid curves- LC vs both control groups, dotted curves– LC vs HD.

Performance of selected miRNA ratios was measured as the ability of miRNA ratios to correctly classify samples from the combined dataset into cancer (SCC and AD) or non-cancer (HD and EB) groups. ROC curves for ratios with area under curve (AUC) over 0.7 are shown in Fig. 6. Only two ratios had AUC of 0.8 or higher – miR-484/miR-222-3p and miR-484/miR-324-5p. Interestingly, the performance of ratios was largely unaffected by the presence of samples from patients with endobronchitis, as indicated by lack of differences between dotted and solid lines. This may suggest that expression profile of endobronchitis may be within the bounds of physiologically normal concentrations at least as the majority of miRNAs retained at this stage of the analysis are concerned.

**Figure 7:**
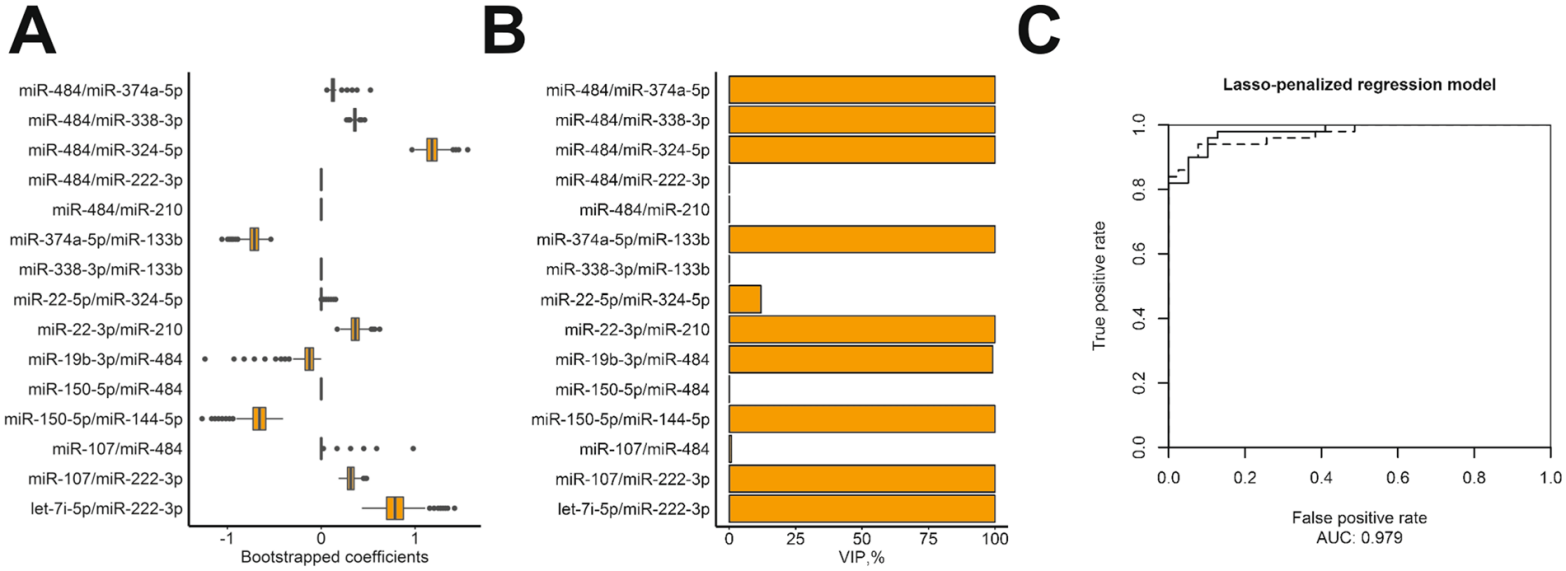
Lasso-penalized bootstrap-enhanced regression of miRNA plasma expression. (**A**) Box plots showing the distribution of regression coefficients for predictors in bootstrap samples; **(B**) Variable importance plot; (**C**) ROC curves for classification of combined dataset using bootstrap-enhanced model (solid), naive non-optimized model (dashed).

Bootstrap-enhanced LASSO-penalized logistic regression on combined data set was performed for miRNA ratios with AUC >0.7 (Fig. 7). The AUC of regression models in bootstrap samples had a mean of 0.9312 (95% CI 0.9305-0.9320) with 1^st^ and 3^rd^ quantiles of 0.9215 and 0.9429, respectively. Variable importance in most bootstrap samples was dominated by 10 miRNA ratios containing 14 miRNAs (miR-22-3p/miR-210, miR-107/miR-222-3p, miR-19b-3p/miR-484, miR-150-5p/miR-144-5p, miR-484/miR-374a-5p, miR-484/miR-338-3p, miR-484/miR-324-5p, let-7i-5p/miR-222-3p, miR-22-5p/miR-324-5p, miR-374a-5p/miR-133b). Lambda corresponding to minimum Brier score was used to fit the final regression model. In the absence of independent validation data, the model was used to predict the classes of the entirety of combined dataset as cancer or non-cancer, yielding an AUC of 0.979.

## Discussion

In this study, we investigated the expression of miRNA in blood plasma of LC patients and cancer-free individuals using miRCURY LNA miRNA qPCR, a platform previously reported to have sensitivity and robustness superior to other widely available platforms, especially in the lower concentration range, which is critical for study of cell-free miRNAs^24^. The results obtained after two-step investigation of miRNA expression further promote the notion of miRNA potential as blood-borne cancer biomarkers. However, out of the enormous amount of miRNA biomarker studies produced to date, only two miRNA panels for diagnosis of lung cancer have successfully proceeded into clinical trials^20,21^. The development of molecular diagnostics involves understanding the biology of the biomarker, including its quantitative and qualitative properties and their dynamics, and developing the analytical techniques tailored to detect the changes of said properties. To date a plethora of issues rooted in both of these areas, including the problems with design, technical reproducibility and credibility of RNA biomarker studies have been identified and discussed, several of which are directly applicable to the current study^27–29^. Our current findings and previous experience suggest a few discussion points, which can be added to this conversation.

Initial premise of the study was to explore the profiles of miRNA expression in SCC and AD, and patients with both diagnoses were included in the discovery profiling. Later all samples of cancer patients were consolidated into a single group. In the course of the study we discovered, that recruiting a matching sample of adenocarcinoma patients has proved harder than expected, because of the high prevalence of smoking-related SCC incidence in local population. Small sample size may explain the observed lack of differences between AD and other groups. Combining the lung cancer subtypes allowed us to expand the sample size and approximate the experimental conditions to the features of the general population. The merging of healthy volunteers and endobronchitis patients into non-cancer group in some analyses also served a similar purpose. Heterogeneity of the screened population should be factored in at some point in the development of diagnostic biomarkers. It can be achieved by narrowing the scope via stratification by a set of clinical or demographic parameters, or by including a wider range of associated conditions, such as inflammatory and non-cancerous diseases, to the study as control groups. To date very few groups have attempted the latter, and most miRNA biomarker studies have chosen the stratification route.

Normalization of expression is another common problem of miRNA studies in biological fluids. So far, no suitably stable “house-keeping” miRNAs have been discovered in blood^30^. Here, to normalize miRNA expression we used ratio-based strategy proposed previously^31^. This approach is helpful, considering the nature of circulating miRNA, but it also creates difficulties for analysis and interpretation of the data. The number of miRNA ratios generated by this approach is high, and thus additional selection steps are required, with the added risk of overfitting and losing valuable data. Interpretation of the relations between the miRNAs in the ratios can also be a complex task. Here we attempted to resolve it by mapping the connections between the selected miRNAs, with the assumption that largest nodes will present the most de-regulated and thus diagnostically significant miRNA markers. However, in some case it may be argued that miRNAs with many connections can also be those with stable expression, while biomarker miRNAs may be outliers.

One major issue we have faced was high variability of miRNA expression both within and between the experiments, which complicates the comparison between cancer and disease-free groups. The extent of variability of known circulating miRNAs and the factors dictating the changes in their expression are still unknown, because the understanding of the mechanisms regulating the entry and clearance of miRNAs in circulation is still vague. Thus, we cannot strictly differentiate between miRNA molecules appearing in the circulation in response to specific events in the tumour and the ones that are associated with physiological shifts and cyclical events during normal functioning of the human body. Unclear is also the effect of each of these factors on the overall concentration of specific miRNA in blood. The procedures used for sampling and processing of plasma, and miRNA extraction can also be a factor, as illustrated by the effects of haemolysis^32^. The reported difference in expression between condition and control groups is often comparable with the combined error of the detection assay (0.5–1.5 Cq), at which point performance of miRNAs as biomarkers is certainly affected. Development of strict inclusion criteria for miRNA samples, based on current analytical techniques and the properties of cell-free miRNA, may be required to tackle this issue. More than anything we feel that our study shows that close attention should be paid to these issues before miRNAs can be adopted as disease markers.

Consistent with other reports, our data has revealed many differences in miRNA expression between the cancer and cancer-free groups. No apparent switch expression of any miRNAs was discovered, as was expected since the expression of only few miRNAs is truly tissue- or organ-specific, while miRNAs found in plasma originate from a variety of locations in the body. However, substantial quantitative differences have been discovered and many of the deregulated miRNAs shown in Table 1 are known to be associated with malignant processes in the lung, acting oncogenes or tumour suppressors.

The miR-19a/b come from a well-known family of oncogenic miRNAs – miR-17-92 cluster, known also as oncomir-1. It was reported to be involved in development of lung cancer by inhibiting apoptosis through targeting PTEN and TP53^33–35^. MiR-19b has been linked to NSCLC and, specifically, squamous cell carcinoma consistent with our current and previous findings. In Boeri *et al*. ratios based on the upregulation of miR-19b were included in the signatures of risk and diagnosis^31^. Both our data and previous research demonstrate that high expression of miR-19b is a characteristic of disease^23,31,36–38^. Another miRNA - miR-324 - has tumour suppressive effects in breast and colon cancers and hepatocellular carcinoma^39,40^. However, it has also been shown to be overexpressed in tumour tissue and plasma of patients with NSCLC, which may indicate its oncogenic role in lung cancer^31,41^. Upregulation of miR-210 in NSCLC tissues was demonstrated in a recent integrative study based on 32 previous profiling studies^42^. It was also repeatedly shown to be upregulated in plasma and serum of lung cancer patients^31,43,44^. The role of miR-222-3p in lung cancer is also unclear. Some reports describe it as an oncogenic miRNA^45^, while others have demonstrated its ability to inhibit growth of lung cancer cells^46^. Its expression in blood has been previously associated with advanced stage of NSCLC and suggested as a marker of lung adenocarcinoma^47,48^. Expression of miR-150 is often deregulated in lung cancer, and it was shown to promote metastasis and proliferation of cancer cells by targeting FOXO4 and SRC kinase signalling inhibitor 1^49–51^. Upregulation of miR-150 expression was associated with poor survival of lung cancer patients and its expression in plasma correlated with the effect of radiation treatment^52,53^. Invasion and proliferation of lung cancer cells is also regulated by miR-15b targeting of TIMP2^54^. It was previously shown to be deregulated in lung tumour tissue in an integrative analysis and suggested as NSCLC marker in serum and plasma^31,55,56^. MiR-140 is a tumour-suppressor^57–59^, previously included in diagnosis signature of lung cancer^31,60^. In contrast to previous reports, our data showed its overexpression in plasma of lung cancer patients. Another tumour suppressor is miR-144, which inhibits proliferation and apoptosis, and regulates glucose metabolism of cancer cells^61–63^. This miRNA is not currently considered as a prospective lung cancer biomarker, but its expression was significantly downregulated in lung cancer patients in our data. Two arms of miR-22 might have opposite roles in lung cancer. The dominant or guide arm - miR-22-3p - acts as tumour suppressor and inhibits growth and metastasis^64,65^. While its passenger strand - miR-22-5p was shown to be elevated in serum of NSCLC, suggesting its possible oncogenic role^66^. In contrast to these reports, miR-22-3p was highly upregulated in lung cancer patients in our study. Lastly, expression of miR-30e was downregulated in lung tumour tissue, and suggested as serum based marker of NSCLC^55,56,67^. Here our data comes into another contradiction to some of the previous reports. A possible explanation for the discrepancies between our data and previous reports may be the different methodologies used to extract and measure miRNA in blood, once gain highlighting the need for unified guidelines for cell-free miRNA analysis. Use of different fractions of blood – plasma or serum, which may carry different pools of circulating miRNAs, such as protein- or vesicle-bound miRNAs, may also contribute to the problem. Finally, another factor can be the general inconsistency of miRNA expression in biological fluids that we have touched upon previously.

Several prominent miRNAs that were deregulated in the discovery set were not included in the further study. For example, miR-486 has previously been shown to be involved in a regulatory pathway along with p53 and another notable miRNA – miR-660^68^. Unfortunately, its plasma levels have also been shown to be susceptible to haemolysis and its downregulation in the lung is unlikely to be detected in blood, as it is easily masked by miRNAs derived from sources other than lung. For instance, it is reportedly expressed in the erythroid progenitor cells and regulates their differentiation^69^. Masking effect can be present for other miRNAs as well, and in this study, we exerted special care when dealing with downregulated miRNAs and mostly concentrated on those that were upregulated. In order to include a downregulated miRNA in a diagnostic panel, it is highly suggested to have comprehensive evidence of its primary tissues of origin in circulation.

Overall, our results are in good correspondence with previous findings and observed plasma miRNA profiles give a good representation of miRNA expression in lung tumours. Based on data from both analysed datasets we propose a 14-miRNA signature with consistently altered expression in plasma for a future validation in extended cohorts of lung cancer patients, patients with non-cancerous lung diseases and control group of cancer- and disease-free individuals.

## Materials and Methods

### Recruitment of patients and study population

Blood samples of 30 healthy individuals were obtained from Center of New Medical Technologies of ICBFM SB RAS (Novosibirsk, Russia) and E.N. Meshalkin Siberian Federal Biomedical Research Center (Novosibirsk, Russia). Samples of 50 non-small cell lung cancer patients with no previous history of cancer were obtained from E.N. Meshalkin Siberian Federal Biomedical Research Center (Novosibirsk, Russia) and Cancer Research Institute of RAMS (Tomsk, Russia). Lung biopsy specimens and imaging techniques were applied to confirm histopathological features and tumour stages of lung cancer patients. None of the patients have undergone surgical treatment or received chemotherapy prior or at the time of blood sampling. Blood samples of 10 patients diagnosed with hyper- or metaplastic endobronchitis were obtained from Cancer Research Institute of RAMS (Tomsk, Russia). Blood samples included in the discovery set were collected between January 2013 and March 2014. Blood samples in verification set were collected between December 2015 and September 2016. Overview of the study population can be found in Table 3.

**Table 3:** Overview of the study population.

Characteristic	Discovery set		Verification set		
	Lung cancer patients	Healthy individuals	Lung cancer patients	Healthy individuals	Endobronchitis patients
	n = 20	n = 10	n = 30	n = 20	n = 10
**Age**					
Mean ± SD	65.0 ± 7.8	51.8 ± 10.0	62.2 ± 5.5	54.3 ± 8.6	61.0 ± 5.4
Range	(41–79)	(31–66)	(55–78)	(42–67)	(57–71)
**Gender**					
Male	20	10	28	18	10
Female	—	—	2	2	—
**Non-smokers**	3	2	2	3	—
**Tumour stage**					
I	1	—	2	—	—
II (A, B)	10	—	14	—	—
III (A, B)	9	—	14	—	—
IV	—	—		—	—
**Tumour subtype**					
Squamous cell carcinoma (SCC)	14	—	28	—	—
Adenocarcinoma (AD)	6	—	2	—	—

All procedures were performed in accordance with the ethical standards of the institutional research committees and with the 1964 Helsinki declaration and its later amendments or comparable ethical standards. Study was approved by ethical committees of ICBFM SB RAS and E.N. Meshalkin Siberian Federal Biomedical Research Center. Full written informed consent was provided by all participants.

### Blood plasma collection and miRNA isolation

Peripheral venous blood was collected in EDTA spray-coated vacutainers (BD, Cat. No. 368589) and processed within 4 hours after blood sampling. To obtain plasma blood was centrifuged at 400 × g for 20 min, supernatant was transferred into a new tube and centrifuged at 800 × g for 20 min. Plasma was collected and stored frozen in aliquots at −80 °C. Only samples without visible signs of haemolysis at all stages of plasma preparation were considered for the study.

To isolate RNA frozen plasma samples were thawed and centrifuged for 5 min at 3,000 × g to rid of the cryoprecipitate. RNA was isolated from the supernatant using single-phase extraction protocol described previously^70^. Briefly, plasma (600 μl) was incubated with single-phase extraction solution, and total miRNA was purified on silica-based spin columns (BioSilica Ltd, Novosibirsk, Russia), divided into two portions corresponding to 480 μl and 120 μl starting plasma volume, and co-precipitated with glycogen. The lesser portion was dissolved in RNase-free water for quality control, while the rest was immediately shipped on dry ice for miRNA profiling.

### Reverse transcription and quantitative TaqMan PCR

Primers and probes for reverse transcription and TaqMan qPCR (Supplementary Table S3) were synthesized in the Laboratory of Medicinal Chemistry (ICBFM SB RAS, Novosibirsk).

Reverse transcription (RT) of miRNA templates was performed as described previously^71^. Each RT reaction was performed in a total volume of 10 μl and contained 3 μl RNA, 50 nM each miRNA-specific primer, 1 unit RiboLock RNAse inhibitor (Thermo Scientific, Lithuania), 100 units MMLV reverse transcriptase (Thermo Scientific, Lithuania), 2 μl 5 × MMLV buffer (250 mM Tris-HCl (pH 8.3 at 25 °C), 250 mM KCl, 20 mM MgCl_2_, 50 mM DTT) (Thermo Scientific, Lithuania), and 250 μM each dNTP. The reaction conditions were as follows: 16 °C–30 min, 42 °C–30 min, 70 °C–10 min. Samples without RNA template and preparations of genomic DNA were used as negative controls.

Each qPCR reaction contained 2.5 μl RT product, 1.25 unit Taq DNA polymerase (BiolabMix, Russia), 3 μl 10 × PCR buffer (750 mM Tris-HCl (pH 8.8 at 25 °C), 200 mM (NH_4_)_2_SO_4_, 0.1% (v/v) Tween 20), 4 mM MgCl_2_, 250 μM each dNTP, 600 nM forward primer, 800 nM universal reverse primer, and 300 nM miRNA-specific TaqMan probe in a total volume of 30 μl. All reactions were performed in duplicates. Real-time PCR amplification was performed with iCycler iQ5 Real-Time PCR Detection System (Bio-Rad, USA).After initial denaturation at 95 °C for 3 min, the reactions were run for 50 cycles at 95 °C for 15 s and 60 °C for 45 s.

### Profiling of miRNA

Profiles of plasma miRNA were obtained using miRCURY LNA miRNA qPCR Panels (Exiqon A/S, Denmark). For profiling in the discovery set an existing Serum/Plasma Panel containing assays for 179 target miRNAs and appropriate quality and technical controls was used to profile 20 LC patients and 10 healthy individuals. For verification set a custom miRNA panel, containing 19 miRNAs selected from the analysis of the discovery data set, was used on 30 LC patients, 20 healthy individuals and 10 patients with endobronchitis. The list of miRNA assays for both data sets can be found in the Supplementary Information (Supplementary Tables S3 and S4).

### Acquisition of TCGA data

Data of miRNA expression in matched tumour tissue and adjacent normal lung tissue samples of lung squamous cell carcinoma patients were retrieved from the publicly accessible data repository at Genomic Data Commons Data Portal (https://portal.gdc.cancer.gov/) using TCGABiolinks R package^72^. The Cancer Genome Atlas Lung Squamous Cell Carcinoma (TCGA-LUSC) project entries of 44 patients containing RPM (reads per million mapped miRNA reads) normalized miRNA-Seq data for 1881 hairpin miRNA precursors (pre-miRNA) and 2027 mature miRNA species (miRNA isoforms quantification data aggregated to mature strands) annotated to miRBase v21 and UCSC were used in the study^73^.

### Statistics and data analysis

For exploratory analyses, miRNAs were normalized to mean Cq of all assays in the sample (global mean). Additionally ratio-based normalization was applied to identify most differently expressed miRNA pairs by subtracting the Cq values of one miRNA from the values of all other miRNAs in that sample without repetitions. Assays detectable in less than 80% of the samples were excluded from the analysis.

Statistical analyses were performed with R (v3.2.3-3.4.2). All tests were considered statistically significant at p < 0.05 unless specified otherwise. Pairwise comparisons were made using T-test (Student or Welch) or one-way ANOVA. Comparison of miRNA expression in matched samples was done using the paired Wilcoxon test. Benjamini-Hochberg FDR correction for multiple testing was applied to all tests.

For bootstrap resampling (2000 iterations) the data set was split 2:1 into training and testing subsets. For fitting of logistic regression models LASSO penalization lambda optimization by 5-fold cross-validation was applied to the training set. The model was then used to predict the values from the respective testing set. Brier score and area under ROC curve were used as measures of model performance.

### Data availability

The dataset generated and analysed in the current study is available in the ArrayExpress repository (accession E-MTAB-6304). Additional data are available in the supplementary files accompanying the manuscript. The data generated in the interim of the study are available from the corresponding author upon reasonable request.

## Electronic supplementary material

Supplementary Dataset 1

Supplementary Dataset 2

Supplementary Information
