# Supplementary material for: Profiling of 179 miRNA Expression in Blood Plasma of Lung Cancer Patients and Cancer-Free Individuals
Source: Sci Rep. 2018 Apr 20;8:6348. doi: 10.1038/s41598-018-24769-2 (PMC5910392; doi:10.1038/s41598-018-24769-2)
Supplement: Supplementary file 3 — Supplementary Information [file 41598_2018_24769_MOESM3_ESM.pdf]

Profiling of 179 miRNA Expression in Blood Plasma of Lung Cancer Patients and Cancer-Free Individuals

Ivan A. ZAPOROZHCHENKO*, Evgeny S. Morozkin, Anastasia A. Ponomaryova, Elena Y. Rykova, Nadezhda V. Cherdyntseva, Aleksandr A. Zheravin, Oksana A. Pashkovskaya, Evgeny A. Pokushalov, Valentin V. Vlassov, Pavel P. Laktionov

*Corresponding author: E-mail: ivanzap@niboch.nsc.ru

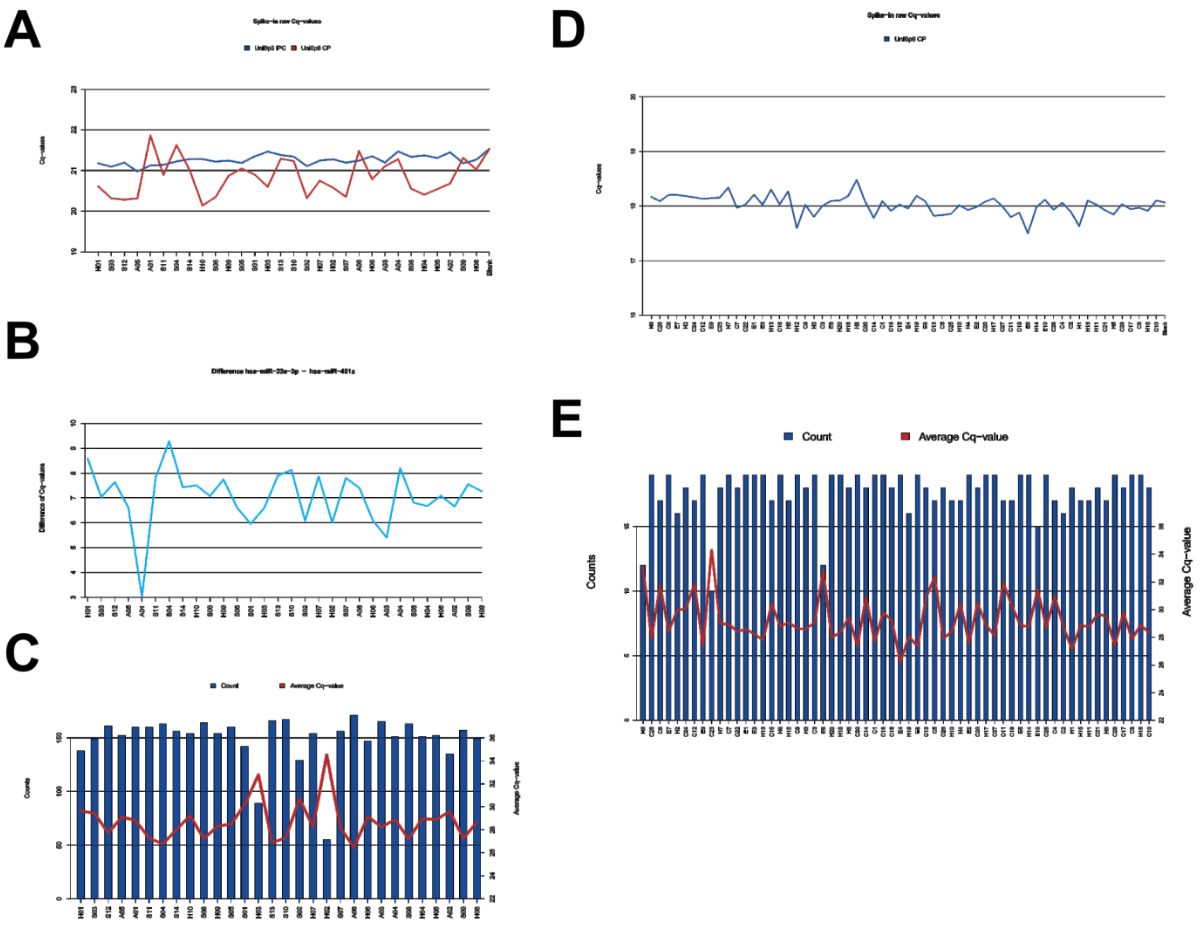

Supplementary Figure S1. Quality control results for discovery (A, B. C) and verification (D, E) sets. A) Amplification of spike-in controls for efficiency of reverse transcription (UniSp6, red) and qPCR (UniSp3, blue) in the discovery set; B) Haemolysis assessment based on the miR-23a – miR-451 difference; C) The miRNA content of the samples in the discovery set. Blue bars show the number of detected essays, red line indicate average Cq-value in the sample (global mean); D) Amplification of spike-in control for efficiency of qPCR (UniSp3, blue) in the verification set; E) The miRNA content of the samples in the verification set. Blue bars show the number of detected essays, red line indicate average Cq-value in the sample (global mean).

Supplementary Table S1. Signatures used for filtering of haemolysis-related miRNAs

**Semi-stringent**	**Stringent**
hsa-miR-16-5p	hsa-miR-16-5p
hsa-miR-92a-3p	hsa-miR-92a-3p
hsa-miR-425-5p	hsa-miR-425-5p
hsa-miR-532-5p	hsa-miR-532-5p
hsa-miR-106a-5p	hsa-miR-106a-5p
hsa-miR-21-5p	hsa-miR-21-5p
hsa-miR-486-5p	hsa-miR-486-5p
hsa-miR-17-5p	hsa-miR-17-5p
hsa-miR-210	hsa-miR-210
hsa-miR-324-3p	hsa-miR-324-3p
hsa-miR-451a	hsa-miR-451a
hsa-miR-652-3p	hsa-miR-652-3p
hsa-miR-126-3p	hsa-miR-126-3p
hsa-miR-320b	hsa-miR-320b
	hsa-miR-22-3p
	hsa-miR-194-5p
	hsa-let-7b-3p
	hsa-miR-140-3p
	hsa-miR-15a-5p
	hsa-miR-15b-3p
	hsa-miR-20a-5p
	hsa-miR-22-5p

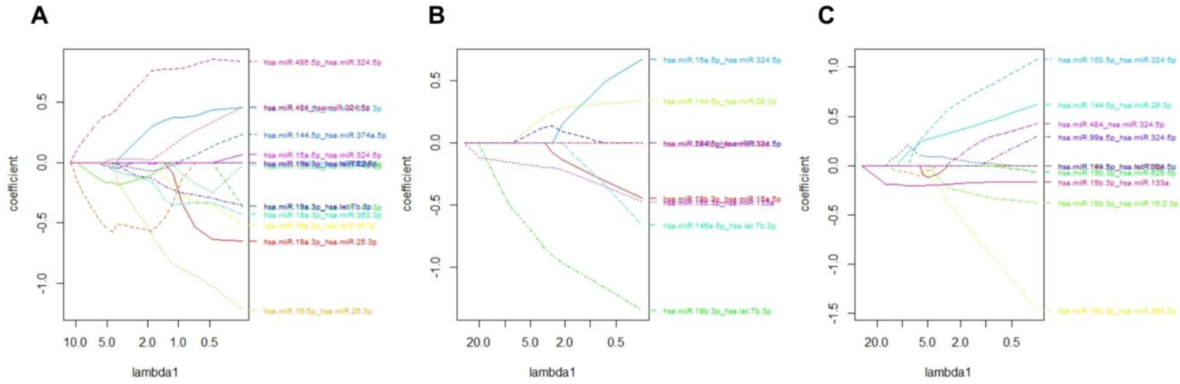

Supplementary Figure S2. LASSO penalized regression models for miRNA ratios with and without filtration of haemolysis-related miRNAs. A) No filtration; B) Semi-stringent haemolysis filtering; C) Stringent haemolysis filtering.

Supplementary Table S2. Sequences of primers and probes used for reverse transcription and TaqMan qPCR

**Universal Reverse Primer**	5’-GTGCAGGGTCCGAGGT-3’
**hsa-miR-16-5p (miR-16)**	RT: 5’-GTCGTATCCAGTGCAGGGTCCGAGGTATTCGCACTGGATACGACCGCCAA-3’<br>Forward: 5’-GCCCGTAGCAGCACGTAAATAT-3’<br>Probe: 5’-(FAM)-GCACTGGATACGACCGCCAA-(FQ)-3’
**hsa-miR-126-3p (miR-126)**	RT: 5’-GTCGTATCCAGTGCAGGGTCCGAGGTATTCGCACTGGATACGACGCATTA-3’<br>Forward: 5’-CCGCTCGTACCGTGAGTAAT-3’<br>Probe: 5’-(FAM)-GCACTGGATACGACGCATTA-(FQ)-3’
**hsa-miR-23a (miR-23a)**	RT: 5’-GTCGTATCCAGTGCAGGGTCCGAGGTATTCGCACTGGATACGACTCAGAC-3’<br>Forward: 5’-CCGCCATTGCACTTGTCTCG-3’<br>Probe: 5’-(FAM)-GCACTGGATACGACTCAGAC-(FQ)-3’
**hsa-miR-451a (miR-451a)**	RT: 5’-GTCGTATCCAGTGCAGGGTCCGAGGTATTCGCACTGGATACGACTCACAA-3’<br>Forward: 5’-CGTCCCTGAGACCCTAACTT-3’<br>Probe: 5’-(FAM)-GCACTGGATACGACTCACAA-(FQ)-3’

Supplementary Table S3. Composition of qPCR panel for discovery set

hsa-miR-19a-3p	hsa-miR-92a-3p	hsa-let-7b-5p	hsa-miR-18a-5p	hsa-miR-532-3p	hsa-miR-339-5p
hsa-miR-19b-3p	hsa-miR-30b-5p	hsa-miR-16-2-3p	hsa-miR-423-3p	hsa-miR-425-3p	hsa-miR-584-5p
hsa-miR-16-5p	hsa-miR-151a-5p	hsa-miR-146a-5p	hsa-miR-629-5p	hsa-miR-28-3p	hsa-miR-204-5p
hsa-miR-25-3p	hsa-miR-378a-3p	hsa-miR-590-5p	hsa-let-7d-3p	hsa-miR-421	hsa-miR-182-5p
hsa-miR-93-5p	hsa-miR-103a-3p	hsa-miR-106b-5p	hsa-miR-17-5p	hsa-miR-33a-5p	hsa-miR-154-5p
hsa-miR-451a	hsa-miR-30c-5p	hsa-miR-652-3p	hsa-miR-140-5p	hsa-miR-146b-5p	hsa-miR-1
hsa-miR-192-5p	hsa-miR-10b-5p	hsa-miR-199a-3p	hsa-miR-374b-5p	hsa-miR-22-5p	hsa-miR-551b-3p
hsa-miR-194-5p	hsa-let-7f-5p	hsa-miR-152	hsa-miR-30a-5p	hsa-miR-93-3p	hsa-miR-501-3p
hsa-miR-32-5p	hsa-miR-181a-5p	hsa-miR-191-5p	hsa-miR-497-5p	hsa-let-7b-3p	hsa-miR-382-5p
hsa-miR-185-5p	hsa-miR-423-5p	hsa-miR-338-3p	hsa-miR-197-3p	hsa-miR-29a-5p	hsa-miR-326
hsa-miR-24-3p	hsa-miR-142-3p	hsa-miR-26b-5p	hsa-miR-155-5p	hsa-miR-210	hsa-miR-10a-5p
hsa-miR-106a-5p	hsa-miR-486-5p	hsa-miR-320b	hsa-miR-339-3p	hsa-miR-27a-3p	hsa-miR-200a-3p
hsa-miR-424-5p	hsa-miR-125b-5p	hsa-miR-99a-5p	hsa-miR-335-5p	hsa-miR-502-3p	hsa-let-7i-3p
hsa-miR-29a-3p	hsa-let-7a-5p	hsa-miR-342-3p	hsa-miR-125a-5p	hsa-miR-128	hsa-miR-361-3p
hsa-let-7i-5p	hsa-miR-126-3p	hsa-miR-23b-3p	hsa-miR-30e-3p	hsa-miR-328	hsa-miR-409-3p
hsa-miR-15a-5p	hsa-miR-150-5p	hsa-miR-139-5p	hsa-miR-99b-5p	hsa-miR-324-5p	hsa-miR-190a
hsa-miR-21-5p	hsa-miR-144-5p	hsa-let-7d-5p	hsa-miR-26a-5p	hsa-miR-141-3p	hsa-miR-127-3p
hsa-miR-148a-3p	hsa-miR-425-5p	hsa-miR-484	hsa-miR-301a-3p	hsa-miR-365a-3p	hsa-miR-543
hsa-miR-148b-3p	hsa-miR-140-3p	hsa-miR-374a-5p	hsa-miR-143-3p	hsa-miR-18a-3p	hsa-miR-766-3p
hsa-miR-27b-3p	hsa-miR-122-5p	hsa-let-7c	hsa-miR-132-3p	hsa-miR-133a	hsa-miR-331-3p
hsa-miR-18b-5p	hsa-miR-215	hsa-miR-22-3p	hsa-miR-136-5p	hsa-miR-223-5p	hsa-miR-29b-2-5p
hsa-miR-223-3p	hsa-miR-130a-3p	hsa-miR-30e-5p	hsa-miR-130b-3p	hsa-miR-376a-3p	hsa-miR-500a-5p
hsa-miR-320a	hsa-miR-29c-3p	hsa-miR-532-5p	hsa-miR-574-3p	hsa-miR-375	hsa-miR-296-5p
hsa-miR-23a-3p	hsa-miR-363-3p	hsa-miR-324-3p	hsa-miR-30d-5p	hsa-miR-885-5p	hsa-miR-485-3p
hsa-miR-107	hsa-miR-186-5p	hsa-miR-205-5p	hsa-miR-28-5p	hsa-miR-95	hsa-miR-605
hsa-miR-101-3p	hsa-miR-15b-3p	hsa-miR-34a-5p	hsa-miR-20b-5p	hsa-miR-200c-3p	hsa-miR-495-3p
hsa-miR-20a-5p	hsa-miR-29b-3p	hsa-miR-145-5p	hsa-miR-195-5p	hsa-miR-20a-3p	hsa-miR-92b-3p
hsa-miR-144-3p	hsa-miR-15b-5p	hsa-miR-151a-3p	hsa-let-7e-5p	hsa-miR-193b-3p	hsa-miR-346
hsa-miR-142-5p	hsa-miR-222-3p	hsa-miR-133b	hsa-miR-199a-5p	hsa-miR-2110	hsa-miR-208a
hsa-let-7g-5p	hsa-miR-660-5p	hsa-miR-221-3p	hsa-miR-505-3p	hsa-miR-106b-3p	

Supplementary Table S4. Composition of qPCR panel for discovery set

**hsa-miR-19b-3p**	**hsa-miR-338-3p**	**hsa-miR-107**	**hsa-miR-133a-3p**
**hsa-miR-25-3p**	**hsa-miR-363-3p**	**hsa-miR-222-3p**	**hsa-miR-210-3p**
**hsa-miR-144-5p**	**hsa-miR-374a-5p**	**hsa-miR-484**	**hsa-miR-215-5p**
**hsa-miR-150-5p**	**hsa-let-7i-5p**	**hsa-miR-22-3p**	**hsa-miR-133b**
**hsa-miR-324-5p**	**hsa-miR-30e-5p**	**hsa-miR-22-5p**	

Color-coding is based on the inclusion criteria (see Results section of the manuscript):

Orange–retained in LASSO models

Green – potentially stable miRNAs

Blue –differently expressed miRNAs

Purple – commonly found in differently expressed ratios
